# Health-Related Quality of Life in Primary Care: Which Aspects Matter in Multimorbid Patients with Type 2 Diabetes Mellitus in a Community Setting?

**DOI:** 10.1371/journal.pone.0170883

**Published:** 2017-01-26

**Authors:** Martina Kamradt, Johannes Krisam, Marion Kiel, Markus Qreini, Werner Besier, Joachim Szecsenyi, Dominik Ose

**Affiliations:** 1 Department of General Practice and Health Services Research; University Hospital Heidelberg, Heidelberg, Germany; 2 Institute of Medical Biometry and Informatics; Department of Medical Biometry; University Hospital Heidelberg, Heidelberg, Germany; 3 Genossenschaft Gesundheitsprojekt Mannheim e.G., Mannheim, Germany; 4 Department of Population Health Sciences, Health System Innovation and Research; University of Utah, Salt Lake City, UT, United States of America; Public Library of Science, FRANCE

## Abstract

**Background and Objective:**

Knowledge about predictors of health-related quality of life for multimorbid patients with type 2 diabetes mellitus in primary care could help to improve quality and patient-centeredness of care in this specific group of patients. Thus, the aim of this study was to investigate the impact of several patient characteristics on health-related quality of life of multimorbid patients with type 2 diabetes mellitus in a community setting.

**Research Design and Methods:**

A cross-sectional study with 32 primary care practice teams in Mannheim, Germany, and randomly selected multimorbid patients with type 2 diabetes mellitus (N = 495) was conducted. In order to analyze associations of various patient characteristics with health-related quality of life (EQ-5D index) a multilevel analysis was applied.

**Results:**

After excluding patients with missing data, the cohort consisted of 404 eligible patients. The final multilevel model highlighted six out of 14 explanatory patient variables which were significantly associated with health-related quality of life: female gender (r = -0.0494; p = .0261), school education of nine years or less (r = -0.0609; p = .0006), (physical) mobility restrictions (r = -0.1074; p = .0003), presence of chronic pain (r = -0.0916; p = .0004), diabetes-related distress (r = -0.0133; p < .0001), and BMI (r = -0.0047; p = .0045).

**Conclusion:**

The findings of this study suggest that increased diabetes-related distress, chronic pain, restrictions in (physical) mobility, female gender, as well as lower education and, increased BMI have a noteworthy impact on health-related quality of life in multimorbid patients with type 2 diabetes mellitus seen in primary care practices in a community setting. The highlighted aspects should gain much more attention when treating multimorbid patients with type 2 diabetes mellitus.

## Introduction

To date the presence of multimorbidity, defined as the co-occurrence of two or more chronic conditions [1], is very common especially among older patients aged 65 years and older [2]. A recent systematic review of observational studies indicated that the prevalence of multimorbidity ranges between 15% to more than 95% in the general population [3]. Especially in patients with diabetes mellitus the presence of additional chronic conditions is typical, so according to the Medical Expenditure Panel Survey of 2004, most adults with diabetes mellitus had at least one (nearly 90%) and about 15% had at least four co-occurring chronic conditions [4]. Multimorbidity in general is known to negatively affect several health outcomes including mortality, hospitalization, and readmission [5] thus leads to greater use and costs of healthcare [6]. Moreover, the presence of multiple co-occurring chronic conditions can complicate care by competing for time, attention, and other resources which might lower quality of healthcare [7].

Recent evidence shows that multimorbidity is becoming the norm [3] and patients with multiple co-occurring chronic conditions account for most consultations in primary care [8]. Thus, GPs and primary care teams have a key role in the management of multimorbid patients [9]. In addition, GPs balance the often competing priorities of each single condition including health and associated everyday problems with a holistic approach [9]. Even though managing multimorbid patients is daily practice for GPs and their teams, they have to face several challenges like coordination and overview on medication to avoid polypharmacy, inadequacy of guidelines and evidence-based medicine for treating patients with multimorbidity, the discrepancy between a fragmented and specialized healthcare system and the needed holistic approach [10–13], as well as the time consumption and burden of contacts, which tend to rise with the number of chronic conditions [14]. Moreover, GPs are forced to consider the needs and wishes of each individual patient in order to provide patient-centered care [15]. From the patients’ perspective, the impact of a disease and treatment on everyday life matters more than the disease by itself [16]. This is one of the reasons why it is so important to consider the improvements in patient-reported health-related quality of life (HRQoL) with treatment, instead of solely relying on changes measured from the healthcare professionals’ perspective like HbA1c-values [17].

HRQoL is a subjective measure and refers to the individual perception of health and those aspects of the multidimensional construct of quality of life that are related to a persons’ health [18–20]. Despite the importance of HRQoL in healthcare, there is still a need to improve care especially for multimorbid patients in primary care [8,10,21,22]. A necessary step in improving healthcare for this group of patients is to understand the association between specific patient characteristics and HRQoL. So, the knowledge of predictors of HRQoL in multimorbid patients may be useful to tailor healthcare according to the individual patient’s needs and to deliver high-quality healthcare. GPs are often in the best position to offer patients support in managing the complexities of their multiple co-occurring conditions [21]. Therefore, an awareness of predictors of HRQoL might be of great interest for GPs, especially in the light of restricted consultation times. For that reason, this study aimed to investigate the impact of various socio-demographic, medical and emotional aspects on patient-reported HRQoL of multimorbid patients with type 2 diabetes mellitus in primary care practices (PCPs) in a community setting.

## Materials and Methods

This analysis is part of a larger trial (GEDIMAplus) and described in detail by Bozorgmehr and colleagues [23]. This study was approved by the ethics committee of the Medical Faculty of the University of Heidelberg, Germany, (S-590/2013) as well as by the ethics committee of the Medical Association Baden-Württemberg, Germany, (B-F-2014-007). All participating patients gave written informed consent. Moreover, the trial is registered with the Current Controlled Trails (ISRCTN 83908315).

### Participants and recruitment

Eligible participants were randomly selected from the overall pool of patients with diagnosed type 2 diabetes mellitus in 21 PCPs located in Mannheim, Germany. All participating PCPs belonged to the PCPnetwork “Genossenschaft Gesundheitsprojekt Mannheim” (GGM) and each of the 32 PCP-teams (physician plus medical assistant) functioned as study center. For the recruitment of patients, PCP-teams were asked to create a list of all potentially eligible patients, which were enrolled in the structured disease management program (DMP) for patients with type 2 diabetes, registered in their practice software in 2013. In Germany, DMPs for selected chronic conditions are embedded in routine primary care and consist of regular follow-up visits every three months including clinical examination, laboratory test and patient education [24,25]. In a next step, PCP-teams selected patients from this list according to the sequences indicated by a previously provided list with random numbers. The randomly selected patients were checked for inclusion and exclusion criteria (Table 1). Each eligible patient should have at least two additional chronic conditions next to type 2 diabetes. These additional chronic conditions included, but were not limited to, atherosclerosis (ICD-10: I70), chronic coronary heart disease (ICD-10: I25), chronic obstructive lung disease (ICD-10: J44), asthma (ICD-10: J45), cerebrovascular diseases (ICD-10: I60—I69), depression (ICD-10: F32 and F33), chronic heart failure (ICD-10: I50), Parkinson’s disease (ICD-10: G20) and/or chronic pain (ICD-10: R52).

**Table 1:** Inclusion and exclusion criteria for possible participants.

Inclusion criteria	Exclusion criteria
At least 18 years of age	Severe acute psychiatric disorders (ICD-10: F20—F29)
Diagnosed type 2 diabetes mellitus (ICD-10: E11- E14)	Dementia (ICD-10: F00—F03)
Enrollment in the DMP for type 2 diabetes	Mental and behavioral disorders due to psychoactive substance use (ICD-10: F11—F16, F18 and F19), except for alcohol (ICD-10: F10) and tobacco use (ICD-10: F17)
At least two additional diagnosed chronic conditions next to type 2 diabetes	Malignant neoplasms (ICD-10: C00—C97) and current chemotherapy or radiotherapy,
	Transplanted organ and/or tissue status (ICD-10: Z94),
	Care involving dialysis (ICD-10: Z49),
	Insurmountable language and communication problems
	Emergency cases

Patients eligible to participate were contacted and invited to participate. This procedure was repeated until approximately 20 patients per PCP-team were recruited. Patients who were willing to participate were informed by their physician about the aims, content, privacy issues and risks related to their participation in the study, and gave their written informed consent.

### Measures

Participating patients were asked to complete a questionnaire regarding their HRQoL (EQ-5D) and their emotional responsiveness specific to diabetes (PAIDshort) as well as additional socio-demographic questions. In addition, physicians completed a questionnaire regarding the patients’ medical attributes (e.g. insulin treatment, HbA1c-value, blood pressure, ICD-10 diagnosis).

The patient-reported HRQoL was measured by the EQ-5D (EuroQol 5-Dimension), a general measure of HRQoL [26]. The EQ-5D classifies an individuals’ health state by asking five ordinal questions regarding mobility, self-care, usual activities, pain/discomfort and anxiety/depression. Each of these dimensions is divided into three levels: no problem, some or moderate problems, extreme problems [27]. The resulting individual health state is then defined by a five-digit number (one number from each of the five dimensions). All EQ-5D health states described by the five-digit number can be additionally converted into a single summary index. Thus, the EQ-5D index is calculated from a profile by applying a formula that attaches weights to each of the five-digit numbers of a health state. The specific set of weights–called value set–is based on a representative sample of the general population of a country or region and generated by elicitation techniques [28,29]. An EQ-5D index of “1” indicates a perfect health state without any impairment of the five determined domains, whereas “0” represents the worst imaginable health state. In this study, the EQ-5D index was generated by converting the EQ-5D health state into a single summary index by applying scores from the European value set [30]. This value set was constructed using data from six European countries (Finland, Germany, The Netherlands, Spain, Sweden, and UK).

A second validated questionnaire was used to measure emotional responsiveness specific to diabetes, called diabetes-related distress. The Problem Areas in Diabetes short-form scale (PAIDshort) is a 5-item questionnaire using a five-point Likert-scale with options ranging from “0” indicating no problems to “4” indicating serious problems [31]. The overall PAIDshort score is calculated as the sum of all 5 items multiplied by 1.25. A minimum overall score of “0” indicates no diabetes-related distress. An overall score of more than “11” indicates an increased negative emotional burden associated with diabetes [32]. Possible reasons for an increased diabetes related-distress include worry about complications, fear of hypoglycemia, major concerns regarding food, exercises and dietary regimes [33].

### Analyses

#### Pre-analysis

In a first step, eligible explanatory variables were identified due to a literature review and an exploratory regression analysis. In addition, variables with more than 100 missing values were excluded from the final analysis. Moreover, the general HRQoL (EQ-5D index) in patients with and without the presence of selected additional chronic conditions next to type 2 diabetes was compared to gain a better conception of the study sample. A linear multilevel regression model with PCP-team as random factor and the respective additional chronic conditions as fixed factors using the restricted maximum likelihood (REML) approach was fitted to compare mean EQ-5D indexes of both groups by calculating p-values for the Type 3 tests of fixed effects. The chosen multilevel approach takes into account patient observation (level one) nested within PCP-teams (level two). Regression coefficients that are allowed to vary randomly across higher level units are called random factors. Whereas fixed factors are defined as regression coefficients that are not allowed to vary randomly across higher level units [34]. The difference between the mean EQ-5D indexes had to be significant (α ≤ 0.05) and the number of participants had to be sufficient to be included as potential explanatory variable (more than 25 participants per group).

#### Multilevel and final descriptive analysis

Due to the hierarchical structure of the data, a linear multilevel regression analysis was applied. This approach takes into account the dependence between patients nested within PCP-teams. For that reason, a series of models were built in sequences to investigate the impact of 14 previous selected potential explanatory variables at patient-level as predictors of HRQoL measured by the EQ-5D index (Table 2). Marital status was dichotomized as married or cohabiting versus other arrangements (separated, single, divorced, widowed) as well as school education which was dichotomized as nine years of school education or less versus at least ten years of school education. Explanatory variables at PCP-team level were not examined. All models consisted of two levels, whereby patients constituted the first level and PCP-teams the second level. Variance partition coefficients in each level were calculated using restricted maximum likelihood (REML). The analysis was completed in numerous stages, whereas each iteration increased the models complexity and therefore explanatory capacity. The multilevel analysis started with an intercept-only model (M1) for the two-level data without predictor variables to calculate the relevant intraclass coefficients (ICC). The intercept-only model was used as reference for comparing the size of contextual variations in EQ-5D in the following models. In the subsequent models socio-demographic aspects (M2), medical aspects (M3), additional chronic conditions (M4) as well as emotional aspects (M5) were included in blocks as fixed effects to examine the amount of additional variance explained by each block of independent variables entered in the model.

**Table 2:** Explanatory variables included in the multilevel analysis.

Variables	Categories/Scoring
**Patient-level**	** **
*Socio-demographic aspects*	
Gender	2 categories: female; male
Age (in years)	Continuous
Marital status	2 categories: married/cohabited; separated/single/divorced/widowed
School education	2 categories: ≤ 9 years; ≥ 10 years
*Medical aspects*	
Number of additional chronic conditions^a^	Continuous (range 2–8): sum of all physician reported additional chronic conditions (ICD-10 code)
Mobility restriction	2 categories: yes; no
Person in need of care	2 categories: yes; no
BMI (kg/m^2^)	Continuous
HbA1c (%)	Continuous
Treatment with insulin	2 categories: yes; no
*Additional chronic conditions*^*a*^	
Chronic heart failure (ICD 10: I50)	2 categories: yes; no
Depression (ICD 10: F32-F33)	2 categories: yes; no
Chronic pain (ICD 10: R52)	2 categories: yes; no
*Emotional aspects*	
Diabetes-related distress (PAIDshort)	Continuous sum score (5 items): 0 (best)-25 (worst)

^a^ next to type 2 diabetes

The coefficients indicate the correlation between the EQ-5D index and each explanatory variable in the final model (M5).

The significance level was set to 5% (two-sided). All descriptive statistical analyses were carried out by using SPSS version 20.0. The multilevel analysis was conducted by using the procedure PROC MIXED in SAS version 9.4.

## Results

### Sample characteristics

The participating 21 PCPs with 32 PCP-teams recruited 495 eligible patients. After excluding participants with missing data a total of 404 patients were included in the final analysis. The mean age of included participants was 67.8 ± 10.8 years, 45.0% (N = 182) were female and 34.2% (N = 138) were treated with insulin (Table 3). The mean EQ-5D index within the study sample was 0.69 ± 0.23.

**Table 3:** Sample description (included patients) with observed means and standard deviation for continuous, and absolute and relative frequencies for categorical variables.

**PCP-level**		
Included PCP-teams	32	
**Patient-level**		
Included patients	404	
EQ-5D index (SD)	0.69	(0.23)
*Socio-demographic aspects*		
Age (SD)	67.80	(10.78)
Gender (female) (%)	182	(45.05)
Marital status (other arrangements than married or cohabited) (%)	161	(39.85)
Years of school education (≤ 9 years) (%)	273	(67.57)
*Medical aspects*		
Additional chronic conditions^a^ (number) (SD)	2.90	(1.02)
Mobility restrictions (yes) (%)	65	(16.09)
Person in need of care (yes) (%)	8	(1.98)
BMI (kg/m^2^) (SD)	31.47	(6.47)
Insulin (yes) (%)	138	(34.16)
HbA1c (%) (SD)	7.17	(1.20)
*Additional chronic conditions*^*a*^		
Chronic heart failure (ICD 10: I50) (yes) (%)	58	(14.36)
Depression (ICD 10: F32-F33) (yes) (%)	65	(16.09)
Chronic pain (ICD 10: R52) (yes) (%)	100	(24.75)
*Emotional aspects*		
Diabetes-related distress (PAIDshort) (SD)	6.20	(6.16)

^a^ next to type 2 diabetes

The multilevel regression model that was fitted to compare mean EQ-5D indexes between participants with and without the presence of selected additional co-occurring chronic conditions next to type 2 diabetes by calculating p-values for the Type 3 tests of fixed effects revealed significant differences for chronic heart failure (ICD 10: I50; *p* < .001), depression (ICD 10: F32-F33; *p* < .001), Parkinson’s disease (ICD 10: G20; *p* < .001) and chronic pain (ICD 10: R52; *p* < .001) (Table 4). A further descriptive analysis of combinations of this additional chronic comorbidities using the above mentioned approach exposed that patients with a combination of depression (ICD 10: F32-F33) and chronic pain (ICD 10: R52) (*p* < .001) as well as a combination of chronic heart failure (ICD 10: I50) and chronic pain (ICD 10: R52) (*p* < .001) differed significantly in both groups (Table 5).

**Table 4:** Description of the EQ-5D index with respect to the presence of a specific comorbidity next to type 2 diabetes. Means unadjusted, p-values adjusted for multilevel structure.

	Yes			No			p-value
	Mean	(SD)	N	Mean	(SD)	N	
Coronary heart disease (ICD 10: I25)	0.71	(0.21)	145	0.67	(0.24)	259	.375
Chronic heart failure (ICD 10: I50)	0.62	(0.25)	58	0.70	(0.23)	346	< .001
COPD (ICD 10: J44)	0.65	(0.22)	53	0.69	(0.24)	351	.161
Asthma (ICD 10: J45)	0.66	(0.24)	27	0.69	(0.23)	377	.973
Depression (ICD 10: F32-F33)	0.62	(0.22)	65	0.70	(0.23)	339	< .001
Parkinson´s disease (ICD 10: G20)	0.11	(0.10)	2	0.69	(0.23)	402	< .001
Cerebrovascular diseases (ICD 10: I60-I69)	0.68	(0.19)	32	0.69	(0.24)	372	.557
Chronic pain (ICD 10: R52)	0.59	(0.26)	100	0.72	(0.22)	304	< .001
Atherosclerosis (ICD 10: I70)	0.63	(0.25)	49	0.69	(0.23)	355	.008

**Table 5:** Description of the EQ-5D index with respect to the presence of the combination of chronic heart failure (ICD 10: I50), depression (ICD 10: F32-F33) and chronic pain (ICD 10: R52) next to type 2 diabetes. Means unadjusted, p-values adjusted for multilevel structure.

	Yes			No			p-value
	Mean	(SD)	N	Mean	(SD)	N	
Chronic heart failure, depression and chronic pain	0.48	(0.42)	2	0.69	(0.23)	402	.088
Depression and chronic pain	0.55	(0.27)	17	0.69	(0.23)	387	< .001
Chronic heart failure and chronic pain	0.41	(0.17)	15	0.70	(0.23)	389	< .001
Chronic heart failure and depression	0.57	(0.21)	9	0.69	(0.23)	395	.098

### Multilevel linear regression model

The intercept-only model (M1) revealed that the greatest proportion of variance in EQ-5D index occurred at the patient-level with 96.8% (ICC = 0.9679), leaving 3.2% of variability to be accounted by for the PCP-team level.

The final multilevel model (MLM) demonstrated that six explanatory patient-level variables were significantly associated with HRQoL measured by the EQ-5D index (Table 6). Each of the regression coefficients presented in the final MLM indicates either a change in the EQ-5D index in comparison to the reference category (categorical variables) or per unit (continuous variables).

**Table 6:** Fixed part results of the random intercept model with overall EQ-5D index as dependent variable (404 patients within 32 PCP-teams), including all variables. Coeff.: regression coefficient, SE: standard error.

	coeff.	(SE)	p-value
Intercept	1.0598	(0.1305)	< .0001
**Patient-level**			
*Socio-demographic aspects*			
Age (year)	-0.0011	(0.0011)	.3217
Gender (female)	-0.0494	(0.0220)	.0261
Marital status (other arrangements than married or cohabited)	-0.0315	(0.0192)	.1023
School education (≤ 9 years)	-0.0609	(0.0175)	.0006
*Medical aspects*			
Additional chronic conditions^a^ (number)	-0.0010	(0.0086)	.9110
Mobility restriction (yes)	-0.1074	(0.0295)	.0003
Person in need of care (yes)	-0.0700	(0.0679)	.3035
BMI (kg/m^2^)	-0.0047	(0.0016)	.0045
Insulin (yes)	0.0148	(0.0222)	.5054
HbA1c (%)	0.0075	(0.0080)	.3524
*Additional chronic conditions*^*a*^			
Chronic heart failure (ICD 10: I50) (yes)	-0.0404	(0.0300)	.1796
Depression (ICD 10: F32-F33) (yes)	-0.0475	(0.0259)	.0682
Chronic pain (ICD 10: R52) (yes)	-0.0916	(0.0253)	.0004
*Emotional aspects*			
Diabetes-related distress (PAIDshort)	-0.0133	(0.0019)	< .0001

^a^ next to type 2 diabetes

Among socio-demographic aspects two significant associations with the EQ-5D index were observed within the variable gender and school education. Female patients had a lower EQ-5D index of 0.0494 compared to male participants (regression coefficient = -0.0494; SE = 0.0011; *p* = .0261) likewise participants with a lower school education level scored lower on the EQ-5D index compared to higher educated participants (regression coefficient = -0.0609; SE = 0.0175; *p* = .0006). Further interesting relationships within medical aspects were discovered. Participants with a mobility restriction had a lower EQ-5D index of 0.1074 (regression coefficient = -0.1074; SE = 0.0295; *p* = .0003) compared to participants without a mobility restriction. Regarding the BMI, each increase of one kg/m^2^ was linked to a 0.0047 decrease of the EQ-5D index (regression coefficient = -0.0047; SE = 0.0016; *p* = .0045). Furthermore, the presence of chronic pain (ICD 10: R52) led to a decrease of the EQ-5D index of 0.0916 compared to participants without (regression coefficient = -0.0916; SE = 0.0253; *p* = .0004). The results regarding emotional responsiveness specific to diabetes showed, that each increase of one point on the PAIDshort scale was linked to a 0.0133 decrease of the EQ-5D index (regression coefficient = -0.0133; SE = 0.0019; *p* < .0001).

All fixed part results of all random intercept models (M1-M5) with overall EQ-5D index as independent variable are presented as supporting information (S1 Table).

**Fig 1:**
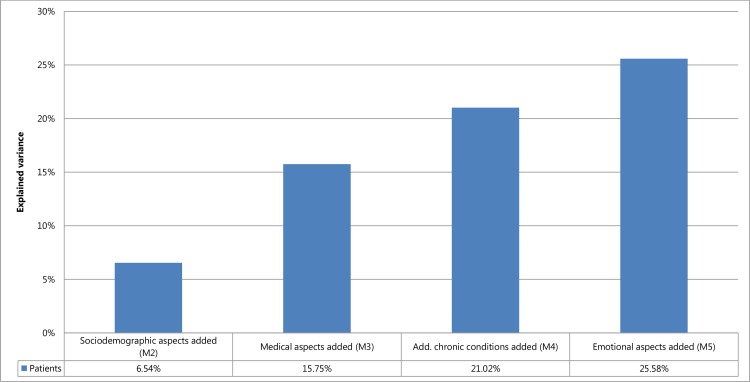
Proportion of variance in overall EQ-5D index explained at the patient-level.

The random part results showed a considerable reduction of observed variance by including explanatory variables at the patient-level in the MLM. Interestingly, by adding each predefined block of variables into the model (M2 = sociodemographic aspects, M3 = medical aspects, M4 = additional chronic conditions, M5 = emotional aspects) a reduction of variance in the EQ-5D index could be observed (S2 Table). A smaller variance proportion means that the added variables explain the variance. Accordingly, the final model explained 25.6% of variance at the patient-level (Fig 1).

## Discussion

The aim of this study was to provide comprehensive data on the impact of various patient characteristics on HRQoL of multimorbid patients with type 2 diabetes mellitus in a community setting. Interesting findings that emerged from the data were the differences of general HRQoL in patients with and without the presence of particular additional chronic conditions next to type 2 diabetes in this specific group of patients. The comparison of patients with and without chronic heart failure (ICD 10: I50), depression (ICD 10: F32-F33), Parkinson’s disease (ICD 10: G20), and chronic pain (ICD 10: R52) as well as a combination of these conditions next to type 2 diabetes revealed significant differences in the EQ-5D indexes between both groups. The study also provides considerable insight to predictors of HRQoL in multimorbid patients. The conducted multilevel analysis highlighted six explanatory patient variables which were significantly associated with HRQoL measured by the EQ-5D index. The female gender, a school education of nine years or less, (physical) mobility restrictions, and the presence of chronic pain were significantly associated with a decrease in HRQoL in multimorbid patients with diabetes mellitus. An increase in diabetes-related distress and BMI was likewise associated with a cutback in HRQoL. However, it is interesting to note that age, the number of chronic conditions, physician-diagnosed depression, insulin treatment, and the HbA1c-value showed no significant association with HRQoL in this group of patients. Taken together, the final MLM was able to explain 25.6% of the variance in HRQoL.

This study exposed an inverse relationship between the presences of multiple co-occurring chronic conditions next to diabetes mellitus and HRQoL. This finding is in good agreement with previous research which stated that additional chronic conditions in patients with diabetes mellitus further lower HRQoL [5,35,36]. Likewise the strong relationship of (chronic) pain and HRQoL has been found to be typical [22,37]. However, the distinction between the impact of different chronic conditions on HRQoL, which were revealed by the results of this analysis, are in contradiction to Heyworth and colleagues [17] findings. Even more surprising was that this study did not support a significant association between HRQoL and physician-diagnosed depression (ICD 10: F32-F33) in the multilevel analysis. In fact, this study only found a significant association between HRQoL and diabetes-related distress, a patient-reported outcome. Likewise, previous studies which found a strong relationship between depressive symptoms and HRQoL in patients with diabetes mellitus focused solely on PROs [38,39]. These findings would appear to indicate that a physician-diagnosed depression has a weaker impact on HRQoL than the patient-reported emotional well-being.

Moreover, this study does not support previous research concerning an important association between the number of chronic conditions and HRQoL [17,39–41]. In fact, contrary to what was previously thought, this study found no significant association between an increasing number of chronic conditions and a decrease in HRQoL.

Another contradiction to previous research, which found evidence for an association between an increase in age and a decrease in HRQoL [39,42], is shown by the results of this study. A possible explanation for this phenomenon is an adaption process called “response shift”, which involves an internal change of standards, values and the conceptualization of HRQoL [43]. So, patients of advanced age as well as patients suffering from chronic conditions over a long period of time may become used to their health status and might downscale their expectations [22].

### Strengths and weaknesses

The analytical approach of a MLM, used in this study to identify predictors of HRQoL, is seen as relevant in health services research due to the hierarchical structure of the patients’ data clustered in PCP-teams [44]. The collection of relevant data for the analysis was done with well-established measures to make sure that the data was valid. In addition, biomedical outcomes like laboratory measures and chronic conditions used in this study were determined by healthcare professionals and not self-reported by patients. Since PCP-teams functioned as study centers and were responsible for recruitment of eligible participants, authorized employees of the study central office conducted practice visits in randomly selected participating PCPs to ensure adherence to the study protocol.

However, a number of potential weaknesses need to be considered. Unfortunately, it was not possible to include Parkinson’s disease as a possible predictor of HRQoL in the multilevel analysis, even if there was a highly significant difference in HRQoL between patients with and without Parkinson’s disease. The cutoff point for inclusion in further analysis was set to more than 25 participants with the presence of a specific additional chronic condition. Thus, Parkinson’s disease was not included as possible explanatory variable due to the fact that the study sample only consisted of 2 patients with Parkinson’s disease. Moreover, within this study the severity of each chronic condition was not assessed. Therefore, it is possible that dissimilar results would have arisen if the focus had been enlarged on the severity of chronic conditions.

Another possible source of weakness is that this study used a convenience sample of PCP-teams. Thus, the motivation of the participating PCP-teams may not be comparable to PCP-teams in general.

### Implications for clinical practice

To date, a fragmented healthcare system and a restricted time-contingent for patient consultations poses a challenge for the delivery of high-quality, patient-centered healthcare. This applies particularly for patients with multiple co-occurring chronic conditions in primary care.

Nowadays, it is widely recognized that the evaluation of appropriate healthcare should not only focus on biomedical outcomes like clinical and laboratory measures. In addition, biomedical outcomes need to be complemented by measures that focuses on patients’ concerns, like how illness and treatment affect their lives [45]. These outcomes are called patient-reported outcomes (PROs) and are based on the direct report of the individual status of a health condition by the patient, without any interpretation by anyone else [46]. One of the most important PROs is HRQoL [16]. Therefore, it is crucial to examine patient-reported HRQoL when determining the quality and patient-centeredness of care.

The diabetes-related distress was measured by the PAIDshort within this study sample and revealed a significant association with HRQoL in the final MLM, whereas healthcare professional-diagnosed depressions did not. This finding might also support the relevance of PROs, above all for chronically ill patients, when examining the well-being of patients as a supplement to physician-reported, biomedical outcomes. For that reason it might be recommended to strengthen the use of an instrument which determines emotional responsiveness specific to diabetes next to capturing HRQoL in research as well as in clinical practice to intensify patient-centeredness in healthcare of multimorbid patients.

The presence of certain chronic conditions next to diabetes mellitus is an additional aspect which might be of great interest when identifying multimorbid patients with an increased chance of a lowered HRQoL. The findings of this study are in line with previous results [22,37] and suggest that HRQoL in patients with diabetes and at least one of these additional chronic conditions: chronic heart failure (ICD 10: I50), depression (ICD 10: F32-F33), Parkinson’s disease (ICD 10: G20), and chronic pain (ICD 10: R52) have a lower HRQoL in general. As a result, physicians should keep an eye on patients, which have a least one of these chronic conditions next to their diabetes.

Accordingly, the findings of this study taken together with earlier research [37,47] can be used to identify multimorbid patients with a higher chance of a lowered HRQoL. A possible reason for a decreased HRQoL could be for example an increased need of additional support in managing the co-occurring conditions and lifestyle changes inherent with the presence of several chronic conditions [48]. Thus, these patients might benefit from improvements in their HRQoL through a reinforced patient-centeredness. In addition, the knowledge of predictors of HRQoL in multimorbid patients in primary care could be used to develop an appropriate set of screening instruments to identify patients more easily which have an increased possibility of a lowered HRQoL. This might help to organize patient consultations more efficiently in primary care according to the individual needs and challenges of each patient [49].

### Conclusion

The findings suggest that age, number of chronic conditions as well as physician-diagnosed depression do not have a significant association with HRQoL in multimorbid patients with type 2 diabetes mellitus seen in PCPs in a community setting. In contrast, increased diabetes-related distress, chronic pain (ICD 10: R52), restrictions in (physical) mobility, female gender, as well as lower education and, increased BMI seem to have a noteworthy impact on HRQoL in this specific group of patients. Thus, the findings highlight, that improvements and patient-centeredness in care might be achieved by giving this aspects much more attention when treating multimorbid patients with type 2 diabetes mellitus. Especially for GPs and primary care teams, these findings might be of great interest because they play a key role in managing this group of multimorbid patients.

## Supporting Information

S1 Table Fixed part results of all random intercept models (M1-M5) with overall EQ-5D index as dependent variable. Coeff.: regression coefficient, SE: standard error.
(PDF)

S2 Table Random part of all five random intercept models with overall EQ-5D index as dependent variable (404 patients within 32 PCP-teams). VC: Variance component; SE: standard error; EV: explained variance.
(PDF)

S1 File Dataset. (XLSX)
