# Supplementary material for: Health-Related Quality of Life in Primary Care: Which Aspects Matter in Multimorbid Patients with Type 2 Diabetes Mellitus in a Community Setting?
Source: PLoS One. 2017 Jan 26;12(1):e0170883. doi: 10.1371/journal.pone.0170883 (PMC5268781; doi:10.1371/journal.pone.0170883)
Supplement: S1 Table — Coeff.: regression coefficient, SE: standard error. (PDF) [file pone.0170883.s001.pdf]

S1 Table. Fixed part results of all random intercept models (M1-M5) with overall EQ-5D index as dependent variable. Coeff.: regression coefficient, SE: standard error.

	**M 1** (N=458)			**M 2** (N=432)			**M 3** (N=432)			**M 4** (N=432)			**M 5** (N=404)		
	coeff.	(SE)	p-value	coeff.	(SE)	p-value	coeff.	(SE)	p-value	coeff.	(SE)	p-value	coeff.	(SE)	p-value
Intercept	0.69	(0.01)	<.00	0.78	(0.06)	<.00	0.99	(0.13)	<.00	0.93	(0.13)	<.00	1.06	(0.13)	<.00
**Patient level**															
Socio-demographic Aspects															
Age (-years)				0.00	(0.00)	.64	0.00	(0.00)	.52	0.00	(0.00)	.29	-0.00	(0.00)	.32
Gender (female)				-0.09	(0.02)	<.00	-0.06	(0.02)	.01	-0.05	(0.02)	.03	-0.05	(0.02)	.03
Marital status (other arrangements than married or cohabited)				-0.04	(0.02)	.08	-0.02	(0.02)	.26	-0.02	(0.02)	.31	-0.03	(0.02)	.10
School education (≤ 9 years)				-0.08	(0.02)	.00	-0.06	(0.02)	.01	-0.06	(0.02)	.00	-0.06	(0.02)	.00
Medical Aspects															
Additional chronic conditions next to type 2 diabetes (number)							-0.02	(0.01)	.12	-0.00	(0.01)	.79	-0.00	(0.01)	.91
Mobility restriction (yes)							-0.14	(0.03)	<.00	-0.12	(0.03)	<.00	-0.11	(0.03)	.00
Person in need of care (yes)							-0.08	(0.08)	.34	-0.06	(0.07)	.45	-0.07	(0.07)	.30
BMI (kg/m²)							-0.01	(0.00)	.00	-0.01	(0.00)	.01	-0.01	(0.00)	.01
Insulin (yes)							0.03	(0.03)	.27	0.03	(0.02)	.22	0.02	(0.02)	.51
HbA1c (%)							-0.01	(0.01)	.56	-0.01	(0.01)	.53	0.01	(0.01)	.35
Additional chronic conditions next to type 2 diabetes															
Chronic heart failure (ICD 10: I50) (yes)										-0.06	(0.03)	.07	-0.04	(0.03)	.18
Depression (ICD 10: F32-F33) (yes)										-0.06	(0.03)	.02	-0.05	(0.03)	.07
Chronic pain (ICD 10: R52) (yes)										-0.10	(0.03)	.00	-0.09	(0.03)	.00
Emotional Aspects															
Diabetes-related distress (PAIDshort)													-0.01	(0.00)	<.00
