# Supplementary material for: Health-Related Quality of Life in Primary Care: Which Aspects Matter in Multimorbid Patients with Type 2 Diabetes Mellitus in a Community Setting?
Source: PLoS One. 2017 Jan 26;12(1):e0170883. doi: 10.1371/journal.pone.0170883 (PMC5268781; doi:10.1371/journal.pone.0170883)
Supplement: S2 Table — VC: Variance component; SE: standard error; EV: explained variance. (PDF) [file pone.0170883.s002.pdf]

S2 Table. Random part of all five random intercept models with overall EQ-5D index as dependent variable (404 patients within 32 PCP-teams). VC: Variance component; SE: standard error; EV: explained variance.

	VC	(SE)	EV
**M1: Intercept-only model**			
Patients	0.0537	(0.0037)	
**M2: Socio-demographic aspects added**			
Patients	0.0502	(0.0039)	6.54%
**M3: Medical aspects added**			
Patients	0.0452	(0.0036)	15.75%
**M4: Additional chronic conditions added**			
Patients	0.0424	(0.0034)	21.02%
**M5: Emotional aspects added**			
Patients	0.0373	(0.0030)	25.58%
